# Indirect neonatal hyperbilirubinemia in hospitalized neonates on the Thai-Myanmar border: a review of neonatal medical records from 2009 to 2014

**DOI:** 10.1186/s12887-018-1165-0

**Published:** 2018-06-12

**Authors:** L. Thielemans, M. Trip-Hoving, J. Landier, C. Turner, T. J. Prins, E. M. N. Wouda, B. Hanboonkunupakarn, C. Po, C. Beau, M. Mu, T. Hannay, F. Nosten, B. Van Overmeire, R. McGready, V. I. Carrara

**Affiliations:** 1 Shoklo Malaria Research Unit, Mahidol-Oxford Tropical Medicine Research Unit, Faculty of Tropical Medicine, Mahidol University, Mae Sot, Thailand; 2 Neonatology-Pediatrics, Cliniques Universitaires de Bruxelles - Hôspital Erasme, Université Libre de Bruxelles, Brussels, Belgium; 3 Centre for Tropical Medicine and Global Health, Nuffield Department of Medicine, University of Oxford, Oxford, UK; 4 Cambodia-Oxford Medical Research Unit, Angkor Hospital for Children, Siem Reap, Cambodia; 5 Angkor Hospital for Children, Siem Reap, Cambodia; 6 University of Groningen, Groningen, The Netherlands; 7 Mahidol-Oxford Tropical Medicine Research Unit (MORU), Faculty of Tropical Medicine, Mahidol University, Salaya, Thailand; 8 University of Glasgow, Glasgow, Scotland UK

**Keywords:** Indirect neonatal hyperbilirubinemia, Jaundice, (LED-) phototherapy, Neonates, Low-resource, Refugee, Migrant, Resource-limited setting, Mortality

## Abstract

**Background:**

Indirect neonatal hyperbilirubinemia (INH) is a common neonatal disorder worldwide which can remain benign if prompt management is available. However there is a higher morbidity and mortality risk in settings with limited access to diagnosis and care. The manuscript describes the characteristics of neonates with INH, the burden of severe INH and identifies factors associated with severity in a resource-constrained setting.

**Methods:**

We conducted a retrospective evaluation of anonymized records of neonates hospitalized on the Thai-Myanmar border. INH was defined according to the National Institute for Health and Care Excellence guidelines as ‘moderate’ if at least one serum bilirubin (SBR) value exceeded the phototherapy threshold and as ‘severe’ if above the exchange transfusion threshold.

**Results:**

Out of 2980 records reviewed, 1580 (53%) had INH within the first 14 days of life. INH was moderate in 87% (1368/1580) and severe in 13% (212/1580). From 2009 to 2011, the proportion of severe INH decreased from 37 to 15% and the mortality dropped from 10% (8/82) to 2% (7/449) coinciding with the implementation of standardized guidelines and light-emitting diode (LED) phototherapy. Severe INH was associated with: prematurity (< 32 weeks, Adjusted Odds Ratio (AOR) 3.3; 95% CI 1.6–6.6 and 32 to 37 weeks, AOR 2.2; 95% CI 1.6–3.1), Glucose-6-phosphate dehydrogenase deficiency (G6PD) (AOR 2.3; 95% CI 1.6–3.3), potential ABO incompatibility (AOR 1.5; 95% CI 1.0–2.2) and late presentation (AOR 1.8; 95% CI 1.3–2.6). The risk of developing severe INH and INH-related mortality significantly increased with each additional risk factor.

**Conclusion:**

INH is an important cause of neonatal hospitalization on the Thai-Myanmar border. Risk factors for severity were similar to previous reports from Asia. Implementing standardized guidelines and appropriate treatment was successful in reducing mortality and severity. Accessing to basic neonatal care including SBR testing, LED phototherapy and G6PD screening can contribute to improve neonatal outcomes.

**Electronic supplementary material:**

The online version of this article (10.1186/s12887-018-1165-0) contains supplementary material, which is available to authorized users.

## Background

Jaundice caused by indirect neonatal hyperbilirubinemia (INH) is a common condition and a frequent cause for admission in health care facilities all around the world [1]. Without timely admission and appropriate management, INH can lead to devastating neurologic disorders [1]. Cerebral palsy, auditory disturbances and gaze abnormalities are classical sequelae of INH [2–4]. Worldwide, 80% of severe INH occurs in resource-limited settings with an estimated mortality rate of 25% and with a 13% risk of developing neurological sequelae [1, 5, 6]. In settings with poor access to care, prematurity and Glucose-6-phosphate dehydrogenase (G6PD) deficiency are important causes of INH [1, 7, 8]. Though phototherapy is a proven and cost effective tool to treat INH, it is not accessible to more than 6 million (~ 45%) of at-risk infants worldwide [9]. Unavailable treatment has clinical, public health, and economic impact for both the health care and education systems [5].The 2013 Lancet report on the Global Burden of Disease added INH to the list of estimated causes of death [10] and it was recognized as an important neonatal condition that deserves global health attention in the post-2015 millennium development goal era [5]. Routine reporting of jaundice data at all health care levels has yet to be implemented as most national records report jaundice incidence rate based on tertiary health care studies or registries. In Asia, the latest national incidence estimates vary widely from 7% in Indonesia, 15% in India, 46% in Myanmar and up to 49% in China [5, 6]. In Myanmar jaundice is the most common reason for private and public hospitals admission of neonates [11]. According to the National Hospital Statistic Report, it is the leading cause of morbidity in neonates (37.8%), responsible for 7.4% of the neonatal mortality [12]. In 2008, in Maela, a refugee camp in Thailand for displaced Myanmar people, Shoklo Malaria Research Unit (SMRU) reported an increasing number of neonates admitted for phototherapy in their newly established neonatal unit once recognition of the condition by the local health staff had improved. By 2011, INH became the most common reason for hospitalization in this particular setting [13] but the characteristics of neonates with INH, burden of severe INH and its associated risk factors were not known. We therefore conducted a retrospective analysis of all medical records of neonates admitted at SMRU clinics between 2009 and 2014 with the aim of addressing this knowledge gap. The objective of this manuscript is to describe the characteristics of neonates with INH, estimate the burden of severe INH and identify factors associated with severity; to develop evidence-based recommendations to further reduce INH morbidity and mortality in the area.

## Methods

This was an analysis of anonymized medical records of neonates born with a gestational age of 28 weeks or more, admitted either at birth or after discharge from the postnatal ward but within 28 days of life to one of the SMRU Special Care Baby Units between January 1, 2009 and December 31, 2014.

### Setting

SMRU is located in Tak province, Northwestern Thailand (Additional file 1). It is an operational field-based research unit combining humanitarian work with research of direct relevance to the local migrant and refugee population. In contrast to refugees, migrants are highly mobile and may have difficulties to access the clinics. SMRU facilities offer basic emergency obstetric and postnatal care; women requiring caesarian section are referred to the nearest Thai hospital within 30–60 min driving time from the clinics. There was no specialized neonatal care facility until 2008 when the first Special Care Baby Unit was established in Maela refugee camp [13], and in 2011, in two additional clinics serving the migrant population. The units provided basic neonatal care including oxygen, intravenous antibiotics, nasogastric feeding and phototherapy. Chest X-ray, assisted ventilation, parenteral feeding and exchange transfusion were not available. Live born neonates with a gestational age below 28 weeks were provided with palliative care [13]. The mortality in this age group approached 100% [14].

Laboratory tests were conducted upon physician request and restricted to blood group testing, hematocrit reading, microscopic examination of urine sediment and cerebrospinal fluid, and serum bilirubin levels (SBR) measurement using a bilirubinometer (Pfaff Medical Bilimeter 2 and 3). Universal G6PD testing of all newborns was not available but the fluorescent spot test [15] was used in cases of INH.

### Clinical approach of INH

**Fig. 1:**
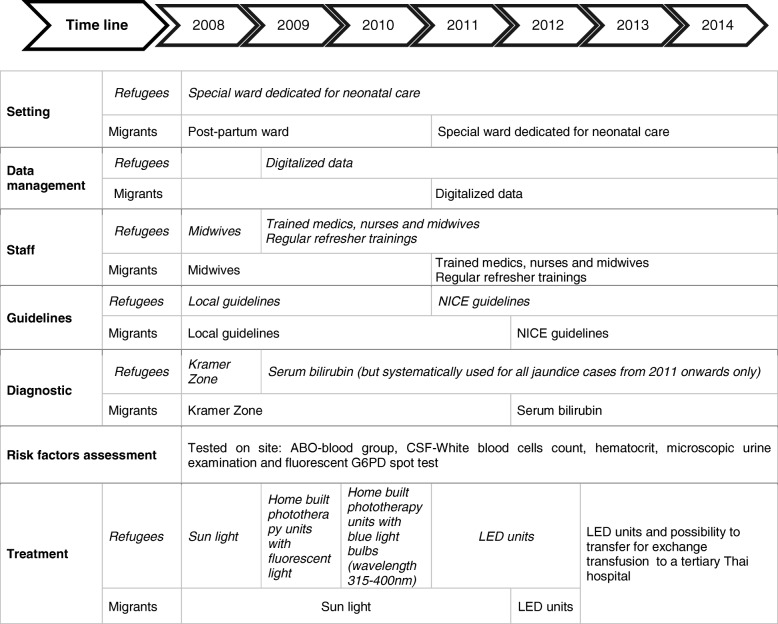
Evolution of care over time. Treatment, diagnostic tools, guidelines and experience of the staff developed over time. Cut off values for phototherapy was based on different guidelines and the type of phototherapy available changed: home built phototherapy units with fluorescent light were available initially and then manufactured bulbs (Philips TL20 W) were used until LED-lights became available. In 2013, collaboration with a tertiary hospital in Thailand was set up to refer neonates who needed exchange transfusion. The condition for referral was a bilirubin more than 550 μmol/L not responding to phototherapy

The decision to use phototherapy was initially based on the Kramer’s scale [16]. Once SBR was available at the clinic, SBR gradually replaced Kramer’s scale as the primary decision tool for treatment. Records with an SBR level were available from 2009 in the refugee clinic and 2012 in the migrant clinics (Fig. 1). Guidelines to start phototherapy have changed over time (Fig. 1) and since 2011 the British National Institute for Health and Care Excellence (NICE) guidelines have been used [17]. Those guidelines base the need for treatment on thresholds varying by gestational age at birth (https://www.nice.org.uk/guidance/cg98/evidence).

Light intensity was routinely measured with a digital lightmeter (Lightmeter by Medical Technology Transfer and Services Ltd) prior to starting phototherapy and the conditions were optimized to have the best intensity possible depending on the type of phototherapy available. Data on light intensity or type of phototherapy used per neonate were not available.

### Jaundice cases classification

Digital records with a diagnosis of jaundice were classified into three categories: i) clinical jaundice without laboratory confirmation (excluded from the analysis), ii) “moderate” INH if at least one SBR value exceeded the phototherapy threshold of the NICE graphs and iii) “severe” INH if at least one SBR value exceeded the SBR exchange transfusion threshold of the NICE graphs. The NICE guidelines did not provide specific recommendations for the treatment of neonates older than 14 days, thus neonates with phototherapy started after 2 weeks of life were excluded from the analysis [17].

### Variables definitions

Relevant variables used for this analysis were birth history, maternal and newborn characteristics, age and diagnosis on admission; additional diagnosis during hospitalization, laboratory results and outcome at discharge.

Primigravida was defined upon registration to antenatal care as first pregnancy (gravidity 1 parity 0). Maternal literacy was defined on the basis of self-reported ability to read. Gestational age was defined by ultrasound [18] or by Dubowitz score [19] for late presenters to the antenatal consultation (after 24 weeks gestation) and classified as very preterm (28 to < 32 weeks), late preterm (32 to < 37 weeks) and term (≥ 37 weeks) following the recommendations of WHO [20].

Instrumental delivery included both vacuum and forceps delivery. Birthweight was considered valid if measured within the first 72 h of life [21, 22] and small-for-gestational-age (SGA) was a birth weight below the 10th percentile of the normal fetal growth reference curve according to Interbio-21 international standards [23]. Every newborn routinely had a surface examination by a staff who had completed a locally developed training course. A standardized newborn examination sheet was completed and any suspected abnormal findings on surface examination and/or auscultation of the praecordium (heart and lungs) was confirmed by a medical doctor [13].

Reported diagnoses of sepsis, meningitis or pneumonia treated with intravenous antibiotics were regrouped into “severe infection”. As laboratory confirmation of these diagnoses was not systematically performed or reported, it was not possible to validate clinically suspected cases. Mild infection was defined as any eye or skin infection treated with oral or topical antibiotics.

Rhesus testing was not available as rhesus incompatibility was deemed very unlikely in this population with very low rates of Rh negative individuals [24]. Coombs test wasn’t available either, thus potential ABO incompatibility was considered for newborns of blood groups A or B born to mothers of blood group O. ABO incompatibility was considered unknown if only one of the pair (mother or neonate) had a known blood group [25].

Outcome at discharge was reported as “alive” or “died”. The total number of livebirths (from 28 weeks’ gestational age) was extracted from SMRU annual reports (http://www.shoklo-unit.com/) and used as denominator for evaluating the changes in proportion of neonates hospitalized with INH.

### Statistical analysis

Statistical analysis was performed using SPSS (IBM SPSS Statistics Version 23, IBM Corporation) and Stata (StataCorp 2015, Version 14.1. College Station, Texas, StataCorp LP) softwares. Categorical variables were described using proportions and compared using the Chi-square test, Fisher’s exact test or Chi-square test for trends; continuous variables were described by their mean and standard deviation and compared using t-test if normally distributed or by their median and interquartile range (IQR) and compared using Mann-Whitney test if non-normally distributed. Binomial or normal 95% confidence intervals (CI) were calculated for proportions or means as appropriate.

Two main outcomes were considered in the analysis: 1) severe INH and 2) late diagnosis (after 72 h of life). Clinical, demographic characteristics and factors associated with each outcome were identified by logistic regression. Univariate Odds Ratio (OR) and 95% CI were generated excluding missing values for a given variable. For each outcome, variables with *p*-values lower than 0.25 in univariable analysis, as well as risk-factors for INH described in the literature, were included in a multivariable model. The final model included all remaining variables with p-values below 0.05 and established risk factors for INH described in the literature.

### Ethics statement

This retrospective analysis of anonymized data was exempted from formal ethical review (confirmed by Oxford Tropical Research Ethics Committee (OxTREC), UK on February 2017) and discussed with the Tak Province Border Community Ethics Advisory Board (T-CAB-01/FEV/2017).

## Results

**Fig. 2:**
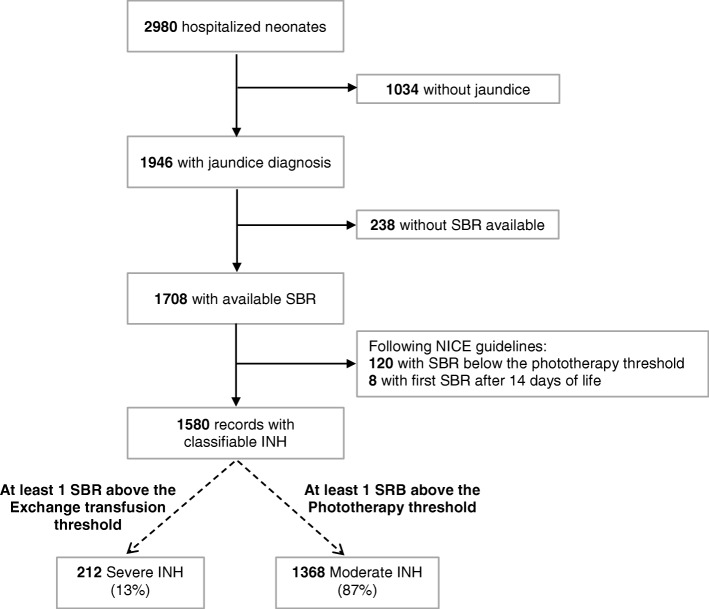
Records of neonates born after 28 weeks of gestational age hospitalized between 2009 and 2014

There were 2980 records of neonates hospitalized between 1st January 2009 and 31st December 2014, representing 23.0% of all live births (*n* = 12,948). Admission within the first 24 h of life contributed to 29.2% (*n* = 871) of hospitalizations. A diagnosis of jaundice was reported in 65.3% (1946/2980) hospitalized neonates of which 87.8% (1708/1946) had at least one SBR value and phototherapy details available. One hundred and twenty records with a maximum SBR level measured below the NICE treatment threshold and eight records with phototherapy started after 14 days of life were excluded. Among the remaining 1580 records, 1368 (86.6%) were classified as moderate INH and 212 (13.4%) as severe INH (Fig. 2). A total of 18,336 SBR measurements in 1580 records were available with a median of 6 SBR measurement [IQR: 3–11] per neonate, ranging from 1 SBR measurement (*n* = 7) to 43 SBR measurement (*n* = 1, recurrent INH). The median SBR value was 249 μmol/l ranging from 24 μmol/l to 1147 μmol/l.

### INH trends

Several changes were observed over time (Table 1). Firstly, the proportion of neonates hospitalized with INH in the refugee population changed significantly; between 2009 and 2011 this proportion was low, ranging between 5.4 and 8.8% of all livebirths, but it increased from 2012 onwards to reach 21.8% of all live births (*n* = 1102) in 2014. Proportions observed in the migrant clinics for the period 2012–2014 increased from 10.6% (134/1270) to 14.6% (209/1430). Overall the proportion of INH in 2014 was 17.7% (Table 1). Secondly, the proportion of neonates hospitalized with INH as sole diagnosis increased from 35.4% in 2009 to 66.4% in 2014. Thirdly, INH was diagnosed 1 day earlier in 2014 compared to 2009 (Table 1) and it became the most common diagnosis among hospitalized neonates from 2012 onwards*.*

**Table 1:** Changes in proportion of neonates hospitalized for indirect neonatal hyperbilirubinemia (INH), INH as sole clinical diagnosis, postnatal age at diagnosis, severity and mortality rate between 2009 and 2014

Time line	2009	2010	2011	2012	2013	2014
Data available						
Refugee site	✓	✓	✓	✓	✓	✓
Migrants sites				✓	✓	✓
SBR available						
Refugee site	✓	✓	✓	✓	✓	✓
Migrants sites				✓	✓	✓
NICE guidelines and LED phototherapy available ^a^			✓	✓	✓	✓
Proportion of NH by total livebirth, n, (%)	82/1520 (5.4)	112/1381 (8.1)	114/1298 (8.8)	364/2573 (14.1)	459/2547 (18.0)	449/2532 (17.7)
NH as sole clinical diagnosis in proportion of total NH ^b^, n (%)	29/82 (35.4)	48/112 (42.9)	61/114 (53.5)	195/364 (53.6)	280/459 (61.0)	298/449 (66.4)
Postnatal age at diagnosis in hours, median, [IQR]	74.5 [48–106]	73.5 [22–122]	67.5 [47–102]	53.5 [37–91]	52 [33–77]	49 [33–81]
Severe INH in proportion of total INH, n (%)	30/82 (36.6)	39/112 (34.8)	17/114 (14.9)	46/364 (12.6)	43/459 (9.4)	37/449 (8.2)
Mortality rate in neonates with severe INH, n (%)	7/30 (23.3)	4/39 (10.3)	2/17 (11.8)	3/46 (6.5)	1/43 (2.3)	0/37 (0.0)

✓Availability of data, SBR, NICE guidelines and LED phototherapy by sites

^a^NICE guidelines and LED phototherapy became available in 2011 for all sites

^b^Clinical diagnoses do not include prematurity, G6PD deficiency or potential ABO incompatibility

The proportion of severe INH among confirmed cases which represented over one third of the confirmed INH in 2009–2010 was reduced by half in 2011 and the decreasing trend persisted until 2014, although at a slower pace (Table 1). Overall the proportion of severe INH in 2014 was 1.5% of all livebirths.

Mortality, among neonates with severe INH, initially 23.3% in 2009, significantly decreased over the years to reach zero in 2014 (Table 1). Mortality rate remained constant and low among neonates with moderate INH.

### General characteristics of neonates with INH

Maternal, obstetric and neonatal characteristics of neonates hospitalized with INH are shown in Table 2. Half of them (52.8%) had a primiparous mother and one third (31.1%) were born preterm (Table 2). INH was the sole diagnosis reported in 57.7% of the records (Table 2). The three most common factors associated with INH among term neonates were G6PD deficiency (219/1088, 20.1%), potential ABO incompatibility (202/1088, 18.6%) and severe infection (202/1088, 18.6%). Ten neonates (0.6%) with INH were referred to the Thai tertiary hospital for further care of whom 4 received exchange transfusion.

**Table 2:** Characteristics of 1580 neonates with indirect neonatal hyperbilirubinemia (INH)

Characteristics	Neonates with INH *n*=1580^a^
Maternal characteristics	
Site, n (%)	
Refugee	1056 (66.8)
Migrant	524 (33.2)
Ethnicity, n (%)	
Karen	1165/1546 (75.4)
Burman	258/1546 (16.7)
Other	123/1546 (8.0)
Age in years, median, (IQR)	24 (20–30) *n* = 1579
Literacy, n (%)	947/1442 (56.7)
Smoking, n (%)	222/1574 (14.1)
Primigravida, n (%)	763 (48.3)
Multiple pregnancy, n (%)	61 (3.9)
Place of birth, n (%)	
SMRU	1423 (90.1)
Tertiary hospital	68 (4.3)
Home	77 (4.9)
Other	12 (0.8)
Type of delivery	
Normal vaginal delivery, n (%)	1427 (90.3)
Breech and face delivery, n (%)	49 (3.1)
Instrumental vaginal delivery, n (%)	62 (3.9)
Caesarian section, n (%)	42 (2.7)
Newborn characteristics	
Gestational age, n (%)	
< 32 weeks	53/1578 (3.4)
32 < 37 weeks	437/1578 (27.7)
≥ 37 weeks	1088/1578 (68.9)
Gender (male), n (%)	922 (58.4)
Small for gestational age, n (%)	297/1554 (19.1)
Congenital abnormality, n (%)	44 (2.8)
Hospitalization characteristics	
INH as sole clinical diagnosis^b^, n (%)	911 (57.7)
Infection	
Severe infection, n (%)	296 (18.7)
Mild infection, n (%)	206 (13.0)
Age in days at admission, median, (IQR)	2 (1–3)
Age in hours at presentation of INH, median, (IQR)	55 [36–92]
Length of stay in days, median, (IQR)	
1–3 days	588 (37.2)
> 3–5 days	307 (19.4)
> 5–8 days	323 (35.1)
> 8 days	362 (39.3)
Mortality during hospitalization, n (%)	31 (2.0)

^a^Denominator unless stated otherwise

^b^Clinical diagnoses do not include prematurity, G6PD deficiency or potential ABO incompatibility

**Fig. 3:**
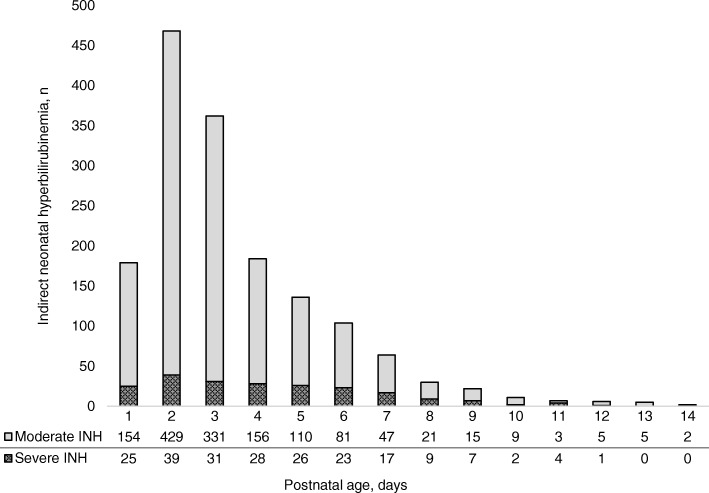
Timing of first serum bilirubin confirming the degree of severity of INH. Each neonate is represented once, when the SBR measurement reached the moderate threshold (and never passed the severe threshold) or reached the severe threshold for the first time in the first 14 days of life

Most INH cases were diagnosed within the first 72 h of life (1009/1580, 63.9%) (Fig. 3). The proportion of neonates with severe INH within the 72 first hours of life was 9.4% (95/1009) and significantly lower (*p* < 0.001) compared to the 20.5% (117/571) of severe INH diagnosed later (> 72 h).

Whilst the proportion of INH cases with G6PD deficiency was equally distributed over the 14 first days of life, there were some striking differences between neonates presenting with an early INH (≤ 72 h of life, *n* = 1009) or late INH (> 72 h of life, *n* = 571) (Additional file 2). After adjustment for other variables, very preterm (< 32 weeks), potential ABO incompatibility and breech or face delivery were independently associated with a diagnosis before 72 h while delivery outside the clinic, severe infection and severe INH were associated with a later diagnosis (Additional file 2).

### Characteristics of neonates with severe INH

Seventy-nine of the 212 (37.3%) neonates with severe INH were first treated for moderate INH at a median postnatal age of 48 h IQR [29–88] and later developed severe INH at a median postnatal age of 97 h IQR [64–136] while 133 (62.7%) presented for the first time with a SBR measurement above the severe threshold line at a median postnatal age of 70 h IQR [33–126].

Factors independently associated with severity after adjustment for variables listed in Table 3 were: very premature (Adjusted Odds Ratio (AOR) 3.3; 95% CI 1.6–6.6) and late premature (AOR 2.2; 95% CI 1.6–3.1); or with a congenital abnormality (AOR 2.4; 95% CI 1.1–5.3). Severe infection (AOR 1.8; 95% CI 1.2–2.7), G6PD-deficiency (AOR 2.3; 95% CI 1.6–3.3) and potential ABO incompatibility (AOR 1.5; 95% CI 1.0–2.2) were also associated to severe INH (Table 3).

**Table 3:** Maternal and newborn characteristics of moderate and severe INH and factors associated with severe INH

Characteristics	Moderate INH, *n*=1368^a^	Severe INH, *n*=212^a^	OR [95%CI]	p-value	AOR^b^ [95%CI]	*p*-value
Maternal characteristics						
Site, n (%)						
Migrant	464 (33.9)	60 (28.3)	1	0.107	–	–
Refugee	904 (66.1)	152 (71.7)	1.3 [0.9–1.8]		–	
Ethnicity, n (%)						
Karen	1014/1348 (75.2)	151/198 (76.3)	1	0.434	–	–
Burman	230/1348 (17.1)	28/198 (13.2)	0.8 [0.5–1.2]		–	–
Other	104/1348 (7.7)	19/198 (9.0)	1.2 [0.7–2.1]		–	–
Age in years, median, (IQR)	24 (20–30)	23 (20–31)	1.0 [0.9–1.0]	0.777	–	–
Literacy, n (%)	846/1280 (66.1)	101/162 (62.3)	0.9 [0.6–1.2]	0.347	–	–
Smoking, n (%)	189/1364 (13.9)	33/210 (15.7)	1.2 [0.8–1.7]	0.477	–	–
Primigravida, n (%)	668 (48.8)	95 (44.8)	0.9 [0.6–1.1]	0.276	–	–
Multiple pregnancy, n (%)	52 (3.8)	9 (4.2)	1.1 [0.5–2.3]	0.758	–	–
Place of birth, n (%)						
SMRU	1241 (90.7)	182 (85.8)	1	0.011	–	–
Tertiary hospital	61 (4.5)	7 (3.3)	0.8 [0.4–1.7]		–	–
Home	58 (4.2)	19 (9.0)	2.2 [1.3–3.8]		–	–
Other	8 (0.6)	4 (1.9)	3.4 [1.0–11.4]		–	–
Breech and face delivery, n (%)	36 (2.6)	13 (6.1)	2.4 [1.3–4.6]	0.008	2.0 [1.0–4.3]	0.056
Instrumental vaginal delivery, n (%)	58 (4.4)	4 (1.9)	0.4 [0.2–1.2]	0.073	–	–
Newborn characteristics						
Gender (male), n (%)	796 (58.2)	126 (59.7)	1.1 [0.8–1.4]	0.731	–	–
Small for gestational age, n (%)	251/1350 (18.6)	46/204 (15.5)	1.3 [0.9–1.8]	0.188	1.3 [0.9–2.0]	0.139
Gestational age, n (%)						
< 32 weeks	38/1367 (2.8)	15/211 (7.1)	3.5 [1.9–6.5]	< 0.001	3.3 [1.6–6.6]	< 0.001
32 < 37 weeks	352/1367 (25.7)	85/211 (40.3)	2.1 [1.6–2.9]		2.2 [1.6–3.1]	
≥ 37 weeks	977/1367 (71.5)	111/211 (52.6)	1		1	
Congenital abnormality, n (%)	34 (2.5)	10 (4.7)	1.9 [1.0–4.0]	0.089	2.4 [1.1–5.3]	0.027
G6PD deficiency, n (%)	213/1364 (15.6)	54 (25.5)	1.9 [1.3–2.6]	< 0.001	2.3 [1.6–3.3]	< 0.001
Potential ABO incompatibility, n (%)	238/1341 (17.7)	45/205 (22.0)	1.3 [0.9–1.9]	0.155	1.5 [1.0–2.2]	0.032
INH as sole clinical diagnosis, n (%)	814 (59.5)	97 (45.8)	1.7 [1.3–2.3]	< 0.001	–	–
Infection, n (%)						
No infection associated	955 (69.8)	123 (58.0)	1	0.003	1	0.006
Associated severe infection	240 (17.5)	56 (26.4)	1.8 [1.3–2.6]		1.8 [1.2–2.7]	
Associated mild infection	173 (12.6)	33 (15.6)	1.5 [0.9–2.3]		1.4 [0.9–2.2]	
Age at presentation, n (%)						
0–24 h	154 (11.3)	37 (17.5)	2.1 [1.4–3.3]	< 0.001	1.5 [0.9–2.4]	0.003
> 24–72 h	160 (55.6)	85 (40.1)	1		1	
> 72 h	454 (33.2)	90 (42.5)	1.8 [1.3–2.4]		1.8 [1.3–2.6]	
Length of phototherapy in hours, median, (IQR)	36 (22–66)	72 (43–133)	–	< 0.001	–	
Mortality during hospitalization, n, (%)	14 (1.0)	17 (8.0)	8.4 [4.1–17.4]	< 0.001	–	–

^a^Denominator unless stated otherwise

^b^Variables included in the final model were: ‘Gestational age’, ‘small for gestational age’, ‘G6PD deficiency’, ‘potential ABO incompatibility’, infection ‘and variables with a p-value < 0.25 in univariable analysis. ‘*NH as sole clinical diagnosis*’ (correlated to ‘*Infection*’) and ‘*Place of birth’* (correlated to *‘Age at presentation’*) were not included in the final model. Only AOR and [95%CI] of known risk factors and of those remaining significant in the final model are reported in this table

The risk of death was 8-times higher in severe INH; 17/212, 8.0% vs. moderate INH; 14/1368, 1.0%, *p* < 0.001 (Table 3) and the risk of INH-related death increased by 3.2 fold (95% CI; 2.1–4.8) with each additional risk factors.

Prematurity was the sole factor reported in 20.3% (43/212) of the neonates with severe INH and severe infection was the most commonly additional factor in preterm neonates (31%, 31/100). G6PD deficiency was the most common factor associated with severe INH among term neonates (32.1%, 36/112), followed by severe infection (22.3%, 25/112) and potential ABO incompatibility (20.5%, 23/112). Overall, the risk to develop severe INH increased significantly with each added risk factor (Table 4).

**Table 4:** Impact of the cumulative number of risk factors on INH severity

Number of risk factors^a^	Moderate INH *N* = 1368	Severe INH *N* = 212	Severity OR [95%CI]
No associated factor, n (%)	523 (38.2)	43 (20.3)	1
One associated factor, n (%)	604 (44.2)	95 (44.8)	1.9 [1.3–2.8]
Combination of 2 factors, n (%)	212 (15.5)	54 (25.5)	3.1 [2.0–4.8]
Combination of 3 or 4 factors, n (%)	29 (2.1)	20 (9.4)	8.4 [4.4–16.1]

^a^The considered risk factors were prematurity, G6PD deficiency, potential ABO incompatibility, severe infection and congenital abnormality

## Discussion

This retrospective analysis confirmed that, with nearly 18% of all livebirths treated for INH, the burden of the disease in this resource-limited area is almost double the worldwide estimates of 10.5% of livebirths that require phototherapy annually [9]. In addition, the high proportion of severe INH and its related mortality contrast with data from high income countries [26] and reinforce the evidence that low-income countries bear the greatest burden of severe INH [1, 26].

The caseload of severe cases was higher in the early years of the Special Baby Care Units while the visual assessment of jaundice was still commonly used. Visual assessment by Kramer zones can be safely used to rule out INH in healthy term neonates if jaundice is limited to the head and torso [27, 28] and might detect severe INH when jaundice has progressed to zones 4 and 5 [29]; however it correlates poorly with measured bilirubin and has limitation for preventing severe INH [28]. The delayed laboratory confirmation likely contributed to the high proportion of severe INH and the higher mortality rate reported during that period.

After the introduction of the NICE guidelines and of LED-light in 2011, the proportion of severe INH was reduced by half despite the increased number of INH cases diagnosed. This suggests the key-role of increased staff awareness and training in using appropriate guidelines, and the effectiveness of the LED-lights. By providing light at the most effective wavelength ranges close to the infant, LED-lights may keep SBR below the severe threshold [30]. These findings confirm those of the study from Myanmar published in 2015 showing that provision of LED-light and staff’s training using standard guidelines reduced severe INH rates drastically [31].

In addition to their impact on the severity of INH these improvements, combined with the possibility to refer for exchange transfusion, had an impact on its mortality which decreased 10-fold between 2009 and 2014.

Apart from prematurity, the three most commonly reported risk factors associated with severe INH in this setting were G6PD deficiency, severe infection and potential ABO incompatibility. They were similar to the risk factors reported previously for low and middle income countries [32].

The prevalence of G6PD deficiency (90% Mahidol variant) in this population is high: 13.7% in adult males [33] and 2–4% in adult females [34]. The increased risk of hyperbilirubinemia in G6PD-deficient neonates might have been further aggravated by an early exposure to naphthalene-containing mothballs used routinely by almost half of the local population [35]. The use of the qualitative fluorescent spot test rather than a quantitative test to diagnose G6PD deficiency in neonates might have underestimated its impact [36]; the G6PD FST has been described not to perform well in neonates possibly due to the higher G6PD activity in neonates then adults [36]. Despite this limitation, G6PD remains significantly associated with severe INH independently of the timing of presentation of INH. Those findings are consistent with those previously described worldwide [17, 37–40].

Potential ABO incompatibility was associated with severity and with an early presentation of INH which is consistent with findings from other studies [32, 41–44]. However, Coombs results were unavailable and the proportion of true ABO alloimmunisation causing INH in this population is still unknown [45, 46].

Nearly a fifth of neonates with INH were treated for a clinically-suspected severe infection. This high proportion of reported infections is consistent with that described in similar low and middle income Asian settings, where 10 to 30% of INH cases are attributed to infections [17, 47]. Its association with the severity of INH might however have been confounded by the similarity of symptoms of a severe infection and of severe INH, and constrained diagnostic laboratory capacity.

Prematurity is an established risk factor for INH [47] but in this particular setting, suboptimal care due to unavailability of parenteral feeding and assisted ventilation, combined with suboptimal visual assessment of jaundice in the preterm neonate [28] might have contributed to the higher rate of severe cases or to the progression from INH to severe INH observed in this setting.

Overall the cumulative effect of risk factors on the risk of severe INH and on the INH-related mortality was significant. This findings support the prediction models based on a combination of risk factors proposed in previous studies [48, 49].

The strength of these results relied on a large dataset of routinely collected clinical and laboratory variables with a low proportion of missing information. The impact of additional factors such as weight loss, bruising or cephalhematoma, having a sibling previously treated for INH, maternal obesity or diabetes, drug-induced labour and rhesus incompatibility were not systematically reported and neither was the intensity or the orientation of the phototherapy lights sources, a limitation of this retrospective design. Those elements should be considered for further evaluation of INH morbidity and mortality in this setting [47, 50].

## Conclusion

The implementation of guidelines for the management of INH, early diagnosis by SBR and treatment with LED phototherapy are three simple and relatively inexpensive tools which have the potential to significantly reduce the number of neonates reaching severe levels of INH.

In a setting where G6PD-deficiency is common, this retrospective evaluation supports the implementation of routine neonatal screening for G6PD deficiency and vigilant observation for jaundice, both in hospital and after discharge home to reduce hospitalizations for severe INH [36, 51]. Finally, although controversies remain on the management of prematurity-associated hyperbilirubinemia and its consequences [52], premature neonates are a vulnerable population group for which the use of usual guidelines might be insufficient. In this population, where prematurity increased 2-fold the risk of severity, the use of safe and efficient prophylactic phototherapy as described by the Cochran neonatal group [53] might be indicated. And it would be worth considering applying the same concept for neonates with cumulating risk factors.

## Additional files

Additional file 1: Location of Shoklo Malaria Research Unit sites. Map of study area showing Shoklo Malaria Research Unit clinic sites: Maela refugee camp, Maw Ker Thai and Wang Pha villages where are located the 2 clinics serving the migrant population (With permission from the Shoklo Malaria Research Unit and Daniel Parker, original copyright 2017) (DOCX 471 kb).

Additional file 2: Factors associated with timing of INH presentation; early INH (≤72 h of life, *n* = 1009) versus late INH (> 72 h of life, *n* = 571) (DOCX 22 kb).

## Abbreviations

AOR: Adjusted Odds Ratio
CI: Confidence interval
FST: Fluorescent spot test
G6PD: Glucose-6-phosphate dehydrogenase
INH: Indirect Neonatal hyperbilirubinemia
INH: Indirect neonatal hyperbilirubinemia
IQR: Interquartile range
LED: Light-emitting diode
NICE: National Institute for Health and Care Excellence
SBR: Serum bilirubin
SGA: Small-for-gestational-age
SMRU: Shoklo Malaria Research Unit
