# Supplementary figures and images for: Indirect neonatal hyperbilirubinemia in hospitalized neonates on the Thai-Myanmar border: a review of neonatal medical records from 2009 to 2014
Source: BMC Pediatr. 2018 Jun 12;18:190. doi: 10.1186/s12887-018-1165-0 (PMC5998587; doi:10.1186/s12887-018-1165-0)

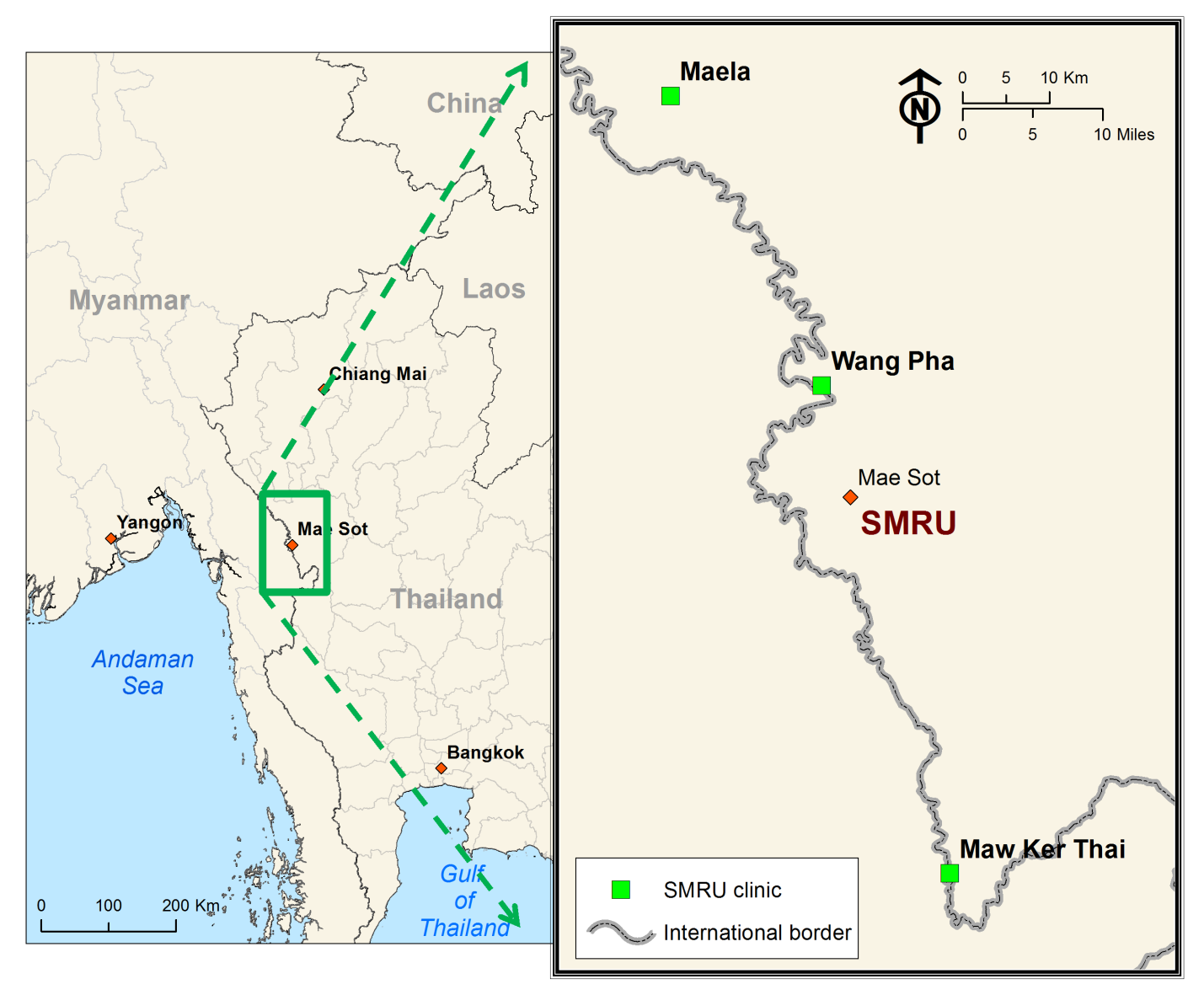

Supplement: Supplementary file 1 — Location of Shoklo Malaria Research Unit sites. Map of study area showing Shoklo Malaria Research Unit clinic sites: Maela refugee camp, Maw Ker Thai and Wang Pha villages where are located the 2 clinics serving the migrant population (With permission from the Shoklo Malaria Research Unit and Daniel Parker, original copyright 2017) (DOCX 471 kb). [file 12887_2018_1165_MOESM1_ESM.docx]
