# Supplementary material for: Indirect neonatal hyperbilirubinemia in hospitalized neonates on the Thai-Myanmar border: a review of neonatal medical records from 2009 to 2014
Source: BMC Pediatr. 2018 Jun 12;18:190. doi: 10.1186/s12887-018-1165-0 (PMC5998587; doi:10.1186/s12887-018-1165-0)
Supplement: Supplementary file 2 — Factors associated with timing of INH presentation; early INH (≤72 h of life, n = 1009) versus late INH (> 72 h of life, n = 571) (DOCX 22 kb). [file 12887_2018_1165_MOESM2_ESM.docx]

**Additional file 2**: Factors associated with timing of indirect neonatal hyperbilirubinemia (INH) presentation; early *(≤72h of life, n=1009)* versus late (>72h *of life, n=571*)

|  | | Univariable analysis | | Multivariable analysis | |
| --- | --- | --- | --- | --- | --- |
| Characteristics | | OR [95%CI] | p-value | AOR* [95%CI] | p-value |
| **Maternal characteristics** | | | | | |
| Site | Refugee | 1.8 [1.0-1.5] | 0.134 | - | - |
|  | Migrant | 1 |  | - |  |
| Ethnicity | Karen | 1 | 0.048 | - | - |
|  | Burman | 0.7 [0.5-0.9] |  | - | - |
|  | Other | 0.9 [0.6-1.4] |  | - | - |
| Literacy | | 1.0 [0.8-1.3] | 0.925 | - | - |
| Smoking | | 1.2 [0.9-1.6] | 0.182 | - | - |
| Primigravida | | 0.8 [0.6-1.0] | 0.030 | - | - |
| Multiple pregnancy | | 2.0 [1.2-3.4] | 0.008 | - | - |
| Place of birth | SMRU | 1 | <0.001 | 1 | <0.001 |
|  | Tertiary hospital | 11.9 [6.0-23.4] |  | 11.9 [5.9-23.9] |  |
|  | Home | 2.0 [1.3-3.2] |  | 1.7 [1.0-2.9] |  |
|  | Other | 4.1 [1.2-13.7] |  | 3.5 [1.0-12.6] |  |
| Breech and face delivery | | 0.4 [0.2-0.8] | 0.003 | 0.3 [0.1-0.8] | 0.012 |
| Instrumental vaginal delivery | | 1.0 [0.6-1.7] | 0.913 | - | - |
| **Newborn characteristics** | | | | | |
| Gender-male | | 1.1 [0.9-1.4] | 0.210 | - | - |
| Small for gestational age | | 0.9 [0.7-1.2] | 0.573 | - | - |
| Gestational age | <32 weeks | 0.4 [0.2-0.8] | 0.037 | 0.4 [0.2-0.8] | 0.045 |
|  | 32 <37 weeks | 1.0 [0.8-1.3] |  | 0.9 [0.7-1.1] |  |
|  | ≥37 weeks | 1 |  | 1 |  |
| Congenital abnormality | | 0.7 [0.3-1.3] | 0.205 | - | - |
| G6PD deficiency | | 1.0 [0.8-1.3] | 0.937 | - | - |
| Potential ABO incompatibility | | 0.6 [0.5-0.8] | <0.001 | 0.6 [0.5-0.8] | 0.002 |
| INH as sole clinical diagnosis | | 1.3 [1.1-1.7] | 0.019 | - | - |
| Infection | No infection associated | 1 | <0.001 | 1 | 0.003 |
|  | Associated severe infection | 1.7 [1.2-2.3] |  | 1.6 [1.1-2.2] |  |
|  | Associated mild infection | 0.9 [0.7-1.2] |  | 0.8 [0.6-1.1] |  |
| Severe INH | | 2.5 [1.9-3.3] | <0.001 | 2.6 [1.9-3.7] | <0.001 |

**The final model includes variables with a p-value< 0.25 in univariable analysis. AOR and [95%CI] of those remaining significant in the final model are presented. A value of OR/AOR above 1 indicates an association with late diagnosis (>72h)*
